# Implementation of an integrative safety consultation service for the use of dietary and herbal supplements among patients with hematological diseases

**DOI:** 10.1007/s00520-025-10227-z

**Published:** 2025-12-04

**Authors:** Ilana Levy Yurkovski, Sohair Bishara-Swaid, Ohad Cohen-Naznin, Yael Gross-Geva, Tamar Elyakim, Samuel Attias, Elad Schiff, Tamar Tadmor

**Affiliations:** 1 Faculty of Medicine, Technion - Israel Institute of Technology, Haifa, Israel; 2 Hematology Institute, Bnai Zion Medical Center, Haifa, Israel; 3 Complementary and Integrative Medicine Service, Bnai Zion Medical Center, Haifa, Israel; 4 Pharmacy, Bnai Zion Medical Center, Haifa, Israel; 5 Internal Medicine B Department, Bnai Zion Medical Center, Haifa, Israel

**Keywords:** Dietary and herbal supplements, Hematology, Interactions, Safety, Complementary medicine, Integrative medicine, Pharmacology, Herb-drug interactions, Natural health products, *Hericium*

## Abstract

**Introduction:**

Dietary and herbal supplements (DHS) are used by 30% of hematological patients despite safety concerns. A service of integrative safety consultations was implemented in the Hematological Institute of Bnai Zion Medical Center. This study examines the effect of such consultation on DHS safety in hematologic patients.

**Methods:**

Patients from the Hematological Institute were referred to the “integrative safety consultation team.” The naturopath recommended DHS according to symptoms disclosed, and safety analysis was performed by the clinical pharmacist. Symptom and side effect assessment was repeated in each follow-up visit to evaluate DHS safety.

**Results:**

Between 2021 and 2024, 42 patients were included. Twenty-eight (67%) used DHS before the consultation. Employed patients were more likely to use DHS (*p* = 0.02). A total of 176 potential interactions were described in 30 patients. Most interactions were theoretical (45%), with a pharmacodynamic additive mechanism (59%), involving herbs (82%) and antihypertensives (26%) or anticoagulants (23%). One side effect was disclosed following *Hericium* prescription to a patient that developed leg edema and neuropathic pain exacerbation (probable causality according to the Naranjo scale and FDA algorithm). Patients’ concerns improved from the first to the second visit. In 21% of the patients who used DHS before the consultation, DHS was documented in the medical chart by the time of first consultation, all of them pertaining to vitamins or minerals.

**Conclusion:**

Consideration of DHS in the management of patients with hematological conditions requires a systematic and comprehensive process to ensure patient safety and wellbeing. An integrative safety naturopathic-pharmacologic consultation can address such needs.

**Supplementary Information:**

The online version contains supplementary material available at 10.1007/s00520-025-10227-z.

## Introduction

Dietary and herbal supplements (DHS) were defined in 1994 by the Dietary Supplement Health and Education Act (DSHEA) as non-tobacco products taken orally but not regulated like drugs by the US Food and Drug Administration (FDA) [1].

More than 50% of the general population and approximately 30% of patients with hematological diseases consume DHS [2, 3].

The use of DHS is associated with safety concerns due to a lack of knowledge and regulation, as well as the risk of side effects and interactions with other medications. In the general population, the incidence of adverse effects related to DHS use is estimated at around 20% [4–6]. A 2017 study of 927 hospitalized patients found a DHS usage rate of 49%, with 47% experiencing potential interactions with medications (15% of which had clinical significance) [7]. Among all patients surveyed, 4% (about 1 in 55 hospitalized patients) experienced a documented adverse event likely related to DHS use [8]. Most reports of DHS interactions are based on in vitro or animal studies, or case reports. In hematologic malignancies, in vitro and animal studies have shown that vitamin C inhibits the cytotoxic activity—and thus the effectiveness—of the proteasome inhibitor bortezomib on malignant plasma cells in multiple myeloma [9].

Drug interactions are especially relevant in patients with hematologic diseases, who often receive anticoagulants, chemotherapy, or biologics [10, 11]. In a study of 157 patients with hematologic malignancies, 734 clinically significant drug-drug interactions were identified per patient-day [12]. For patients taking anticoagulants, both older drugs like warfarin and newer anticoagulants were associated with an increased risk of interactions [13]. These studies did not specifically assess DHS interactions, which are less studied in this population.

Simultaneously, it is well established that DHS may alleviate symptoms related to hematological diseases or their treatments [3], particularly in mitigating side effects from treatments for hematologic malignancies such as lymphoma [14]. For these reasons, clear, evidence-based guidelines are required to ensure the safe and effective use of DHS in patients with hematologic conditions.

Since 2018, the Hematology Institute at Bnai Zion Medical Center has operated an integrative safety clinical consultation service (naturopathic-pharmacologic) for DHS use.

The current study aims to evaluate the impact of such integrative safety consultation on the safety of DHS use among patients with hematological conditions.

## Methods

### Study design

This was a prospective descriptive implementation study conducted at the Hematology Institute of Bnai Zion Medical Center in Haifa, Israel, between 2021 and 2024. The institute provides outpatient consultations and treatments, with approximately 8000 annual visits for patients with a variety of hematologic diseases, including hemato-oncology, benign hematology, and coagulation disorders.

### Ethics

The study was approved by the institutional ethics committee of Bnai Zion Medical Center in accordance with the Declaration of Helsinki (Approval No. 0149–20-BNZ) and registered at www.clinicaltrials.gov (NCT05982262).

### Study population

**Fig. 1:**
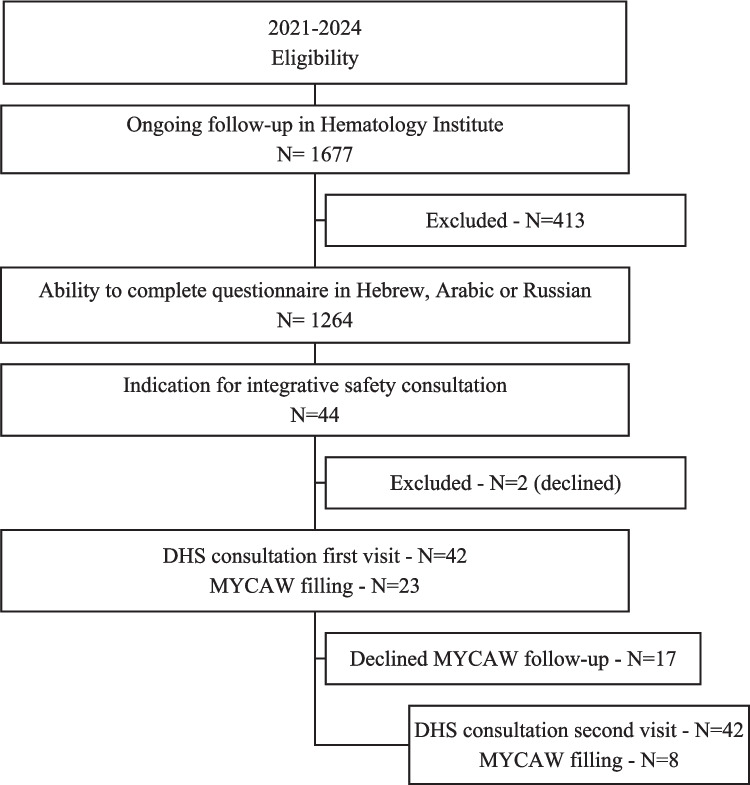
Recruitment algorithm

Patients were referred for integrative safety (naturopathic-pharmacological) consultations by the medical or nursing staff of the Hematology Institute, based on the following inclusion criteria: (1) ongoing follow-up for a hematologic diagnosis; (2) ability to complete questionnaires in Hebrew, Russian, or Arabic; and (3) an indication for consultation, such as (a) patient request for more information on DHS use, (b) current DHS use, (c) desire to start DHS use, (d) interest in learning about DHS for their health condition, or (e) a recommendation from the medical team to use DHS for symptom relief or to improve adherence to conventional treatment. No exclusion criteria were applied. After receiving an explanation on the study, patients provided written informed consent. Recruitment algorithm is presented in Fig. 1.

### Intervention

The integrative safety (naturopathic-pharmacological) consultation included a naturopath and a clinical pharmacist, with documentation recorded in the electronic medical record. Copies of the consultation were provided to the patient, the hematologist, the naturopath, and the pharmacist. Each consultation involved at least two visits.

#### First visit

Patients were asked to document all DHS they were using or intended to use. Socio-demographic and clinical data were collected through patient history and medical record review, with special attention to chronic medications (e.g., anticoagulants, chemotherapy, biologics). Efficacy assessments were performed by the naturopath. Patients who consented completed the Measure Yourself Concerns and Wellbeing (MYCAW) questionnaire to further assess symptoms. Safety assessments were conducted by the clinical pharmacist using three scientific databases (Natural Medicine Comprehensive Database [15], Lexicomp drug interactions [16], and Memorial Sloan Kettering Cancer Center “About Herbs” [17]). Each potential interaction was classified as pharmacokinetic (involving absorption, distribution, metabolism, or elimination) or pharmacodynamic (antagonism, additive, or synergism) and research basis (theoretical, in vitro, animal, or human studies) was reported by the clinical pharmacist according to the mentioned databases. Based on these data and clinical judgement, decision was taken by the clinical pharmacist, the naturopath, and the treating physician, to discontinue/not recommend the DHS or to specifically monitor patient during therapy. The recommendation to initiate, discontinue, or continue specific DHS was documented in the medical record.

#### Follow-up visits

The second visit occurred in person or virtually at least 1 week after the initial consultation. Additional follow-ups were conducted as needed, based on recommendations from the medical or nursing staff, the patient, the naturopath, or the pharmacist. Follow-up visits included reassessment of DHS safety and efficacy, with patients completing follow-up MYCAW questionnaires if they consented.

### Training and quality control

The consultations were conducted by two naturopaths with a minimum of 5 years of clinical experience in oncology or hemato-oncology. The clinical pharmacist held a PharmD degree with expertise in DHS. A five-member data safety monitoring board reviewed the intervention’s safety and data quality annually.

### Outcomes

The primary outcome was DHS safety, assessed by the number of moderate to severe potential interactions prevented through consultation and the frequency of DHS-related adverse events. The Naranjo scale and the adapted FDA causality algorithm were used to assess adverse event causality [18]. Only events rated as “possibly related” or higher were considered relevant.

The secondary outcome was symptom improvement as measured by MYCAW scores before and after consultation. MYCAW consists of two stages: patients define one or two main concerns and rate their severity on a 0–6 scale. During follow-ups, patients re-rate these concerns without seeing prior scores. MYCAW was chosen for its validity, multilingual availability, and suitability for integrative medicine research.

An additional secondary outcome was patient-provider communication, assessed by documentation of DHS use in medical records before and after consultation.

### Sample size calculation

Based on previous research estimating DHS-related adverse events at 18% [11], with about 50% of these related to drug interactions, we hypothesized a 10% adverse event rate for DHS prescribed post-consultation. Assuming a 95% confidence level, 5% alpha, and 10% precision, a minimum sample size of 35 participants was calculated using Scalex and ScalaR software.

### Statistical analysis

Data were analyzed using IBM SPSS version 22. Descriptive statistics (mean, standard deviation, median, range, incidence) were calculated for demographic and clinical variables. For independent group comparisons, unpaired *t*-tests were used for normally distributed continuous variables, Mann-Whitney tests for non-normally distributed variables, and chi-square or Fisher’s exact tests for categorical variables. Paired *t*-tests and Wilcoxon signed-rank tests were used for within-patient comparisons before and after intervention. All tests were two-tailed, with statistical significance set at *p* < 0.05.

## Results

### Socio-demographic and clinical data

Between 2021 and 2024, 42 patients were included in the study, with a median age of 64 years and 27 (68%) women. Twenty-eight patients (67%) reported using DHS before the consultation, primarily vitamins (21 patients, 50%), minerals (12 patients, 29%), or herbs (12 patients, 29%) as detailed in Tables 1 and 2. DHS use was more common among employed patients (43% vs. 0%, *p* = 0.02). No other significant differences were found in baseline characteristics between DHS users and non-users (Tables 1 and 2).

**Table 1:** Use of dietary and herbal supplements by socio-demographic characteristics

Characteristic	Total (*N* = 42)	DHS users (*N* = 28)	Non-DHS users (*N* = 14)	*p*-value
Age (years) — mean (SD)	63 (10.3)	61 (9.1)	64 (11.7)	0.27
Female sex	27 (68%)	19 (68%)	8 (57%)	0.36
Marital status				0.53
Married	35 (75%)	22 (79%)	13 (93%)	
Divorced	3 (6%)	2 (7%)	1 (7%)	
Widowed	3 (6%)	3 (11%)	0	
Single	1 (2%)	1 (4%)	0	
Employment status				0.02
Independent	9 (19%)	4 (14%)	5 (36%)	
Employed	12 (25%)	12 (43%)	0	
Unemployed	1 (2%)	0	1 (7%)	
Housewife	2 (4%)	2 (7%)	0	
Retired	18 (38%)	10 (36%)	8 (57%)

**Table 2:** Use of dietary and herbal supplements by medical characteristics

Characteristic	Total (*N* = 42)	DHS users (*N* = 28)	Non-DHS users (*N* = 14)	*p*-value
Hematologic diagnosis				0.36
Lymphoma	16 (34%)	8 (29%)	8 (57%)	
Multiple myeloma	15 (32%)	11 (39%)	4 (29%)	
Chronic lymphocytic leukemia	6 (13%)	6 (21%)	0	
Chronic myeloid leukemia	4 (9%)	3 (11%)	1 (7%)	
Myelodysplastic syndrome or acute leukemia	2 (4%)	1 (4%)	1 (7%)	
Comorbidities				
Number of comorbidities — mean (SD)	2.2 (2.2)	2.4 (2.1)	2.1 (2.6)	0.74
Neurological	16 (34%)	10 (36%)	6 (43%)	0.45
Cardiovascular	16 (34%)	11 (39%)	5 (36%)	0.55
Endocrine/metabolic	15 (32%)	12 (43%)	3 (21%)	0.15
Rheumatologic	6 (13%)	3 (11%)	3 (21%)	0.31
Non-hematologic malignancy	4 (9%)	3 (11%)	1 (7%)	0.59
Oncologic treatment				
Monoclonal antibodies	12 (26%)	10 (36%)	2 (14%)	0.14
Immunomodulators	8 (17%)	6 (21%)	2 (14%)	0.46
Proteasome inhibitors	5 (11%)	3 (11%)	2 (15%)	0.51
Tyrosine kinase inhibitors	4 (9%)	3 (11%)	1 (7%)	0.59
BCL-2 inhibitors	3 (6%)	2 (7%)	1 (7%)	0.72
Chemotherapy	6 (13%)	5 (18%)	1 (7%)	0.33
Other medications				
Number of other medications — mean (SD)	5.0 (3.7)	4.9 (3.7)	5.3 (4.2)	0.78
Anticoagulants	20 (43%)	12 (43%)	8 (57%)	0.57
Steroids	13 (28%)	9 (32%)	4 (29%)	0.74
Antidiabetic medications	7 (15%)	5 (18%)	2 (14%)	0.73
Antihypertensives	5 (11%)	14 (50%)	6 (43%)	0.67

### Safety

A total of 176 potential interactions of moderate to severe clinical significance were identified in 30 patients (71%) during the consultation, averaging 4.2 clinically significant interactions per patient. Of these, 54 interactions (31%) were associated with pre-existing DHS use (Supplement 1), while 122 (69%) were related to DHS recommended by the naturopath (Supplement 2). Most involved herbs (145 interactions, 82%) and primarily affected antihypertensive drugs (46 interactions, 26%) or anticoagulants (41 interactions, 23%). As outlined in Table 3, when comparing interactions with pre-existing DHS from newly recommended DHS, we noted a higher rate of vitamins, minerals, and non-vitamin/mineral supplements in pre-existing DHS as opposed to more herbs and mushrooms in recommended DHS (*p* < 0.001), more chemotherapy involved in interactions with pre-existing DHS and more anticoagulants and antidiabetic medications involved in interactions with recommended DHS (*p* < 0.001), more pharmacokinetic interactions in pre-existing DHS and more pharmacodynamic interactions in recommended DHS (*p* = 0.009), more in vitro and less animal studies in pre-existing DHS (*p* = 0.001). Finally, more pre-existing DHS involved in interactions were discontinued (*p* = 0.001). Patients using a higher number of DHS were more likely to have potential interactions (*p* = 0.03), especially with herbs (*p* = 0.01) or non-vitamin/mineral supplements (*p* = 0.03). No significant differences in interaction risk were observed based on other patient characteristics (Table 4).

**Table 3:** Description of potential interactions with dietary and herbal supplements

Characteristics	Interactions with pre-existing DHS	Interactions with newly recommended DHS	*p*-value
Number of potential interactions	*N* = 54	*N* = 122	
Involved supplements			**< 0.001**
Vitamins and minerals	2 (4%)	0	
Non-vitamin/mineral supplements	9 (17%)	0	
Herbs	36 (67%)	109 (89%)	
Mushrooms	1 (2%)	13 (11%)	
Other	6 (11%)	0	
Involved medications			**< 0.001**
Chemotherapy	18 (33%)	1 (1%)	
Biologic agents	1 (2%)	7 (6%)	
Antihypertensives	12 (22%)	34 (28%)	
Anticoagulants	6 (11%)	35 (29%)	
Antidiabetic medications	0	15 (12%)	
Proton pump inhibitors	4 (7%)	6 (5%)	
Statins	3 (6%)	3 (2%)	
Other	10 (19%)	21 (17%)	
Interaction type			**0.009**
*Pharmacokinetic*			
Absorption	0	0	
Metabolism	21 (39%)	31 (25%)	
Excretion	0	0	
*Pharmacodynamic*			
Additive/synergistic	23 (43%)	81 (66%)	
Antagonistic	10 (19%)	10 (8%)	
Research basis			**0.001**
Theoretical	22 (41%)	57 (47%)	
In vitro	24 (44%)	22 (18%)	
Animal studies	6 (11%)	37 (30%)	
Human studies	2 (4%)	6 (5%)	
Pharmacist/physician recommendation			**0.001**
Discontinue/do not recommend supplement	16 (30%)	11 (9%)	
Monitoring required	38 (70%)	111 (91%)

**Table 4:** Frequency of potential interactions by patient characteristics

Characteristic	Potential interactions (*N* = 30)	No potential interactions (*N* = 12)	*p*-value
Age (years) — mean (SD)	62 (11)	65 (8.0)	0.40
Female sex	21 (70%)	6 (50%)	0.19
Marital status			0.16
Married	27 (90%)	8 (67%)	
Divorced	1 (3%)	2 (17%)	
Widowed	2 (7%)	1 (8%)	
Single	0	1 (8%)	
Employment status			0.25
Independent	5 (16%)	4 (33%)	
Employed	9 (30%)	3 (25%)	
Unemployed	0	1 (8%)	
Housewife	1 (3%)	1 (8%)	
Retired	15 (50%)	3 (25%)	
Hematologic diagnosis			0.34
Lymphoma	12 (40%)	4 (33%)	
Multiple myeloma	12 (40%)	3 (25%)	
Chronic lymphocytic leukemia	3 (10%)	3 (25%)	
Chronic myeloid leukemia	2 (7%)	2 (17%)	
Myelodysplastic syndrome or acute leukemia	1 (3%)	1 (8%)	
Comorbidities			
Neurological	14 (47%)	2 (17%)	0.07
Cardiovascular	11 (37%)	5 (42%)	0.51
Endocrine/metabolic	9 (30%)	6 (50%)	0.19
Rheumatologic	4 (13%)	2 (17%)	0.56
Non-hematologic malignancy	2 (7%)	2 (17%)	0.32
Oncologic treatment			
Monoclonal antibodies	9 (30%)	3 (25%)	0.53
Immunomodulators	6 (20%)	2 (17%)	0.59
Proteasome inhibitors	3 (10%)	2 (17%)	0.46
Tyrosine kinase inhibitors	2 (7%)	2 (17%)	0.32
Chemotherapy	5 (17%)	1 (8%)	0.43
Other medications			
Number of other medications — mean (SD)	5.3 (3.9)	4.0 (3.3)	0.33
Anticoagulants	16 (53%)	4 (33%)	0.18
Steroids	10 (33%)	3 (25%)	0.26
Antidiabetic medications	6 (20%)	1 (8%)	0.20
Antihypertensives	16 (53%)	4 (33%)	0.18
Dietary and herbal supplements			
Total number of DHS — mean (SD)	2.6 (2.9)	1.2 (1.0)	**0.03**
Number of vitamins	0.8 (0.9)	0.8 (1.0)	0.88
Number of minerals	0.4 (0.7)	0.3 (0.5)	0.65
Non-vitamin/mineral supplements	0.4 (1.0)	0.0 (0.0)	**0.03**
Number of herbs	0.7 (1.1)	0.1 (0.3)	**0.01**
Other supplements	0.1 (0.3)	0.0 (0.0)	0.38

### Adverse events

One adverse event (2.3%) occurred despite the consultation. A 59-year-old male patient, in remission from aggressive lymphoma after chemotherapy and biologics, was taking gabapentin for grade 2 chemotherapy-induced peripheral neuropathy. After a safety review found no documented interactions between gabapentin and *Hericium erinaceus* (lion’s mane mushroom), this DHS was recommended to alleviate neuropathy symptoms. Within a week, the patient developed non-pitting edema in the legs and worsening neuropathic pain. A comprehensive workup found no other cause, and symptoms resolved within 2 weeks of discontinuing the mushroom supplement. Causality was rated as “probable” by both the modified Naranjo scale and FDA algorithm.

### Symptoms and concerns

**Fig. 2:**
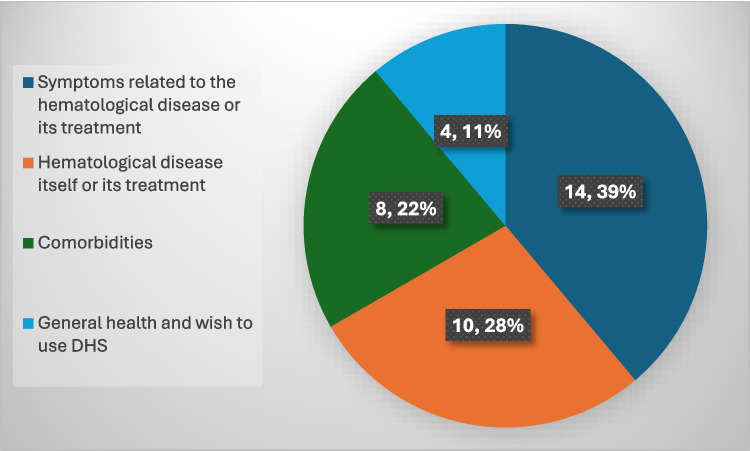
Distribution of MYCAW results by categories

**Fig. 3:**
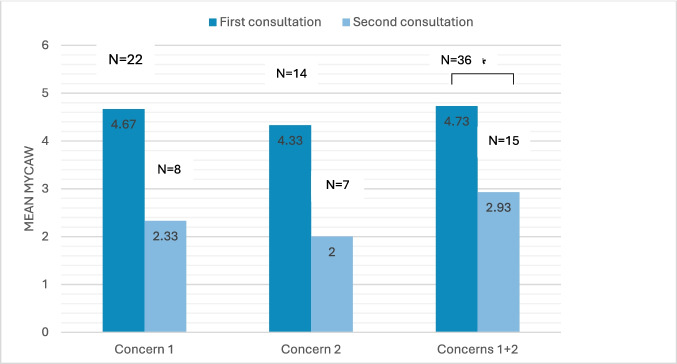
Comparison of average MYCAW in first and second consultations

Of the 23 patients (55%) who completed the MYCAW questionnaire, concerns were categorized into four groups: (1) disease- or treatment-related symptoms (e.g., pain, fatigue, constipation) (14 issues, 41%), (2) the hematologic disease itself or its treatment (10 issues, 29%), (3) comorbid conditions (e.g., hypertension, kidney failure) (8 issues, 24%), and (4) general health and desire for DHS (4 issues, 12%) (Fig. 2). Only 8 patients completed a follow-up MYCAW, limiting comparative analysis. Furthermore, because MYCAW is an individualized tool in which each patient defines their own concerns, missing responses cannot be validly imputed. Therefore, and considering low statistical power, results should be interpreted as exploratory feasibility data. For the primary concern, MYCAW scores improved from 4.67 ± 1.37 (*n* = 22) to 2.33 ± 1.21 (*n* = 8), though not statistically significant (*p* = 0.052). For the secondary concern, scores improved from 4.33 ± 1.51 (*n* = 14) to 2.0 ± 1.67 (*n* = 7) (*p* = 0.065). When averaging both concerns, a statistically significant improvement was observed from 4.73 ± 1.34 (*n* = 36) to 2.93 ± 2.02 (*n* = 15) (*p* = 0.008) (Fig. 3).

### Patient-physician communication

Of the 28 patients who reported DHS use during consultation, only 6 (21%) had DHS use documented in their medical records prior to the intervention, all involving vitamins or minerals.

## Discussion

This study provides valuable insights into the importance and necessity of integrative safety consultations for patients with hematologic malignancies.

The high rate of DHS use (67%) observed in our study is notable, exceeding that reported in previous studies [3]. This may be due to selection bias, as patients who were interested in DHS or referred for consultation were more likely to participate, potentially overrepresenting DHS users. Another possible explanation is the thorough questioning by the consultation team, which may have uncovered more DHS use compared to previous studies. Future research should include validated questionnaires and structured interviews to accurately assess DHS usage, similar to studies conducted in hospitalized patients [7].

The differences in DHS use across socioeconomic groups (higher usage among employed patients) highlight the need for a deeper understanding of the factors influencing DHS use in this population. The most common supplements used were vitamins, minerals, and herbs, consistent with a previous study on cancer patients, where vitamins and minerals were the most frequently used supplements [19].

The identification of 176 potential moderate to severe interactions in 71% of patients in the Hematology Institute, aligns with findings from oncology studies [20]. However, similar research in hematologic diseases is scarce. Of the DHS evaluated, 15% were discontinued or not recommended, while 85% required close monitoring. This underscores the critical importance of professional consultation, as provided in this study through the combined expertise of the naturopath and clinical pharmacist. This finding is supported by a 2019 study describing an algorithm-based information system for identifying pharmacokinetic interactions between DHS and cancer treatments [21]. Such systems emphasize the growing need for advanced tools to manage potential interactions, especially as DHS use continues to rise among patients with hemato-oncological conditions.

The contrasting interaction patterns between pre-existing and newly recommended DHS highlight the different contexts in which these supplements were used. Pre-existing DHS, chosen independently by patients and often without professional input, were more heterogeneous and more frequently involved pharmacokinetic interactions with chemotherapy, underscoring the potential risks of unsupervised DHS use during hematologic treatment. In comparison, the DHS newly recommended during the integrative consultation were primarily herbs and mushrooms and were associated mainly with pharmacodynamic interactions, most of which could be safely managed through structured monitoring, illustrating how guided integrative care can mitigate rather than amplify safety risks.

Interestingly, 69% of interactions were related to DHS suggested by the naturopath, highlighting the importance of collaboration between naturopaths and clinical pharmacists. The naturopaths in this study had substantial experience in the hemato-oncology setting, emphasizing the need for ongoing communication between integrative and conventional care teams to balance safety with patient wellbeing—a core principle of integrative medicine. This point also calls for broader implementation of integrative consultations in public healthcare systems, including coverage by national health insurances, to make these services accessible to patients from lower socioeconomic backgrounds or remote areas.

Most interactions were theoretical, with only 5% supported by human studies. This highlights the need for prospective research in hemato-oncology to better understand DHS mechanisms and interactions with commonly used drugs. Despite the hematologic context and frequent use of biologics and chemotherapy, the most common interactions were with antihypertensives and anticoagulants, mirroring findings from a review that highlighted the interaction risks of DHS with cardiovascular drugs [22]. Although only one adverse event was reported, the potential for harm remains even after careful safety reviews, emphasizing the need for long-term monitoring and evidence-based guidance for DHS use in hematologic cancer care [20].

The MYCAW score improvements suggest potential symptom relief from DHS use, consistent with preliminary research showing symptom improvement in lymphoma patients [14]. Overall and due to limited statistical power, one should look for general direction and clinical meaning of MYCAW variation. Indeed, a threshold of 2-point MYCAW reduction that was reached in the current study has been considered clinically meaningful in former studies [23]. However, the small number of follow-up MYCAW respondents limits the strength of this finding which should be interpreted as exploratory feasibility data, reinforcing the need for larger, well-designed trials.

Finally, the low documentation rate of DHS use in medical records (21%), even though this exceeded the 11% reported in previous inpatient studies [7], underscores the need for educational interventions for healthcare providers, especially in hematology clinics. Encouraging non-judgmental patient-provider communication about DHS, including systematic questioning, documentation, and safety assessments, could bridge this gap.

### Limitations

Despite its valuable findings, this study has limitations. First, reporting bias may have influenced the high prevalence of DHS use, as patients were specifically questioned about supplements in a structured manner; this likely increased disclosure compared with routine hematology visits. Second, selection bias is inherent to the referral process, which was driven by patient interest, clinician concern, or a desire for guidance regarding DHS. As a result, the sample may not reflect the broader hematology population and may overrepresent individuals motivated to discuss or use DHS. Third, the limited number of follow-up MYCAW responses restricts interpretation of efficacy outcomes, which should be viewed as exploratory feasibility data rather than evidence of clinical effectiveness.

## Conclusions

In summary, while DHS use is common among patients with hematologic malignancies and may offer symptom relief, the high potential for interactions with conventional treatments highlights the need for systematic integrative safety naturopathic-pharmacological consultations. Based on our findings, we recommend embedding an integrative physician or clinical pharmacist in every hematology clinic to assess DHS safety and guide usage. Future research should focus on larger, multicenter prospective studies to better understand the safety and efficacy of integrative interventions in hematology care.

## Supplementary Information

Below is the link to the electronic supplementary material.

ESM 1 Supplementary Material 1 (DOCX 20.5 KB)

ESM 2 Supplementary Material 2 (DOCX 25.2 KB)

## Data Availability

No datasets were generated or analysed during the current study.
