# Supplementary material for: Implementation of an integrative safety consultation service for the use of dietary and herbal supplements among patients with hematological diseases
Source: Support Care Cancer. 2025 Dec 4;34(1):6. doi: 10.1007/s00520-025-10227-z (PMC12678462; doi:10.1007/s00520-025-10227-z)
Supplement: Supplementary file 1 — Supplementary Material 1 (DOCX 20.5 KB) [file 520_2025_10227_MOESM1_ESM.docx]

Supplement 1: List of potential interactions with pre-existing DHS

| **DHS** | **Drug** | **Potential effect** | **Research basis** | **Recommendation** |
| --- | --- | --- | --- | --- |
| Alpha lipoic acid | Levothyroxin | Impairment of drug effect | Animal | Monitor |
| Alpha lipoic acid | Aspirin | Increased risk of bleeding | Theoretical | Monitor |
| Cat's claw | R-CHOP | Decreased immunosuppression | In vitro | Discontinue |
| Cat's claw | R-CHOP | Increased drug blood level | In vitro | Discontinue |
| Cordyseps | Apixaban | Increased risk of bleeding | Theoretical | Monitor |
| Cratageus | Bisoprolol | Hypotension | Theoretical | Monitor |
| Curcumin | Allopurinol | Hepatotoxicity | In vitro | Discontinue |
| Curcumin | Atorvastatin | Hepatotoxicity | In vitro | Discontinue |
| Curcumin | DA-EPOCH | Hepatotoxicity | In vitro | Discontinue |
| Curcumin | Mirena | Increased drug blood level | Theoretical | Monitor |
| Curcumin | Atorvastatin | Increased drug blood level | Theoretical | Discontinue |
| Curcumin | Fentanyl | Increased drug blood level | Theoretical | Discontinue |
| Curcumin | DA-EPOCH | Increased drug blood level | Theoretical | Discontinue |
| Curcumin | Venetoclax | Increased drug blood level | Theoretical | Discontinue |
| Curcumin | Amlodipine | Increased drug blood level | Theoretical | Discontinue |
| Curcumin | DA-EPOCH | Impairment of drug effect | Animal | Discontinue |
| Curcumin up | R-CHOP | Decreased immunosuppression | In vitro | Monitor |
| Curcumin up | R-CHOP | Hepatotoxicity | In vitro | Monitor |
| Curcumin up | R-CHOP | Impairment of drug effect | In vitro | Monitor |
| Curcumin up | R-CHOP | Increased drug blood level | In vitro | Monitor |
| Curcumin up | R-CHOP | Immunosuppression | Animal | Monitor |
| Ginkgo biloba | Estradiol | Impairment of drug effect | In vitro | Discontinue |
| Ginkgo biloba | Lansoprazole | Impairment of drug effect | In vitro | Discontinue |
| Melatonin | Gabapentin | Increased sedative effect | In vitro | Monitor |
| Melatonin | Omeprazole | Increased drug blood level | In vitro | Monitor |
| Melatonin | Gabapentin | Impairment of drug effect | In vitro | Monitor |
| Melatonin | Aspirin | Increased risk of bleeding | Theoretical | Monitor |
| Melatonin | Bisoprolol | Hypotension | Human | Monitor |
| Melatonin | Ramipril | Hypotension | Human | Monitor |
| Neta lady | Bisoprolol | Increased drug blood level | In vitro | Monitor |
| Neta lady | Omeprazole | Increased drug blood level | In vitro | Monitor |
| Neta lady | Atorvastatin | Increased drug blood level | Animal | Monitor |
| Neta lady | Aspirin | Increased risk of bleeding | Theoretical | Monitor |
| Neta lady | Bisoprolol | Hypotension | In vitro | Monitor |
| Neta lady | Ramipril | Hypotension | In vitro | Monitor |
| Omega 3 | Aspirin | Increased risk of bleeding | In vitro | Discontinue |
| Panax ginseng | R-CHOP | Decreased immunosuppression | In vitro | Discontinue |
| Panax ginseng | R-CHOP | Increased drug blood level | In vitro | Discontinue |
| Pomegranate | Trazodone | Increased drug blood level | Theoretical | Monitor |
| Pomegranate | Cadesartan | Hypotension | Theoretical | Monitor |
| Pomegranate | Lercanidipine | Hypotension | Theoretical | Monitor |
| Prostate health | R-CHOP | Decreased immunosuppression | Animal | Monitor |
| Prostate health | R-CHOP | Increased drug blood level | Animal | Monitor |
| Resveratrol | Trazodone | Increased drug blood level | Theoretical | Monitor |
| Resveratrol | Esomeprazole | Increased drug blood level | Theoretical | Monitor |
| Resveratrol | Apixaban | Increased risk of bleeding | Theoretical | Monitor |
| Resveratrol | Cadesartan | Hypotension | Theoretical | Monitor |
| Resveratrol | Lercanidipine | Hypotension | Theoretical | Monitor |
| Spirulina | DA-EPOCH | Decreased immunosuppression | Theoretical | Monitor |
| Taurine | Bisoprolol | Hypotension | Theoretical | Monitor |
| Vitamin C | R-CHOP | Impairment of drug effect | In vitro | Monitor |
| Vitamin D | Dexamethasone | Impairment of drug effect | Theoretical | Monitor |
