# Supplementary material for: Implementation of an integrative safety consultation service for the use of dietary and herbal supplements among patients with hematological diseases
Source: Support Care Cancer. 2025 Dec 4;34(1):6. doi: 10.1007/s00520-025-10227-z (PMC12678462; doi:10.1007/s00520-025-10227-z)
Supplement: Supplementary file 2 — Supplementary Material 2 (DOCX 25.2 KB) [file 520_2025_10227_MOESM2_ESM.docx]

Supplement 2: List of potential interactions with newly recommended DHS

| **DHS** | **Drug** | **Potential effect** | **Research basis** | **Recommendation** |
| --- | --- | --- | --- | --- |
| Achilea milefolium | Aspirin | Increased risk of bleeding | In vitro | Monitor |
| Aloe vera | Aspirin | Increased risk of bleeding | In vitro | Monitor |
| Andrographis paniculata | Aspirin | Increased risk of bleeding | In vitro | Monitor |
| Andrographis paniculata | Amlodipine | Hypotension | Animal | Monitor |
| Angelica sinensis | Aspirin | Increased risk of bleeding | Animal | Monitor |
| Astragalus | Cyclophosphamide | Decreased immunosuppression | Animal | Monitor |
| Astragalus | Prednisone | Decreased immunosuppression | Animal | Monitor |
| Atractylodes Macrocephala | Aspirin | Increased risk of bleeding | In vitro | Monitor |
| Bitter orange | Fluticasone | Increased drug blood level | In vitro | Monitor |
| Boswelia serulata | Mirena | Increased drug blood level | Theoretical | Monitor |
| Bupleurum chinensis | Montelukast | Increased drug blood level | In vitro | Monitor |
| Bupleurum chinensis | Aspirin | Increased risk of bleeding | In vitro | Monitor |
| Bupleurum chinensis | Bisoprolol | Hypotension | Human | Monitor |
| Bupleurum chinensis | Metformin | Hypoglycemia | Animal | Monitor |
| Bupleurum chinensis | Ramipril | Hypotension | Human | Monitor |
| Cinnamoum verum | Atorvastatin | Hepatotoxicity | Human | Monitor |
| Cinnamoum verum | Insulin | Hypoglycemia | Theoretical | Monitor |
| Cinnamoum verum | Metformin | Hypoglycemia | Theoretical | Monitor |
| Cinnamoum verum | Amlodipine | Hypotension | Animal | Monitor |
| Citrus aurantium | Duasteride | Increased drug blood level | In vitro | Monitor |
| Citrus aurantium | Esomeprazole | Increased drug blood level | In vitro | Monitor |
| Citrus aurantium | Oxybutynin | Increased drug blood level | In vitro | Monitor |
| Codonopsis | Aspirin | Increased risk of bleeding | Theoretical | Monitor |
| Coix lacryma | Insulin | Hypoglycemia | Theoretical | Monitor |
| Cordyceps sinensis | Aspirin | Increased risk of bleeding | Animal | Monitor |
| Cordyceps sinensis | Clopidogrel | Increased risk of bleeding | Animal | Monitor |
| Cordyceps sinensis | Pomalidomide | Impairment of drug effect | Animal | Monitor |
| Crataegus | Apixaban | Increased risk of bleeding | Animal | Monitor |
| Crataegus | Aspirin | Increased risk of bleeding | In vitro | Monitor |
| Crataegus | Clopidogrel | Increased risk of bleeding | Animal | Monitor |
| Crataegus | Bisoprolol | Hypotension | Theoretical | Monitor |
| Crataegus | Cadesartan | Hypotension | Animal | Monitor |
| Crataegus | Ramipril | Hypotension | Theoretical | Monitor |
| Crataegus | Lercanidipine | Hypotension | Animal | Monitor |
| Cynara | Esomeprazole | Increased drug blood level | In vitro | Monitor |
| Ehamannia glutinosa | Bortezomib | Increased drug blood level | Theoretical | Not recommended |
| Ganoderna lucidum | Insulin | Hypoglycemia | Theoretical | Monitor |
| Ganoderna lucidum | Metformin | Hypoglycemia | Theoretical | Monitor |
| Ganoderna lucidum | Aspirin | Increased risk of bleeding | Theoretical | Monitor |
| Ganoderna lucidum | Amlodipine | Hypotension | Animal | Monitor |
| Ganoderna lucidum | Losartan | Hypotension | Theoretical | Monitor |
| Ganoderna lucidum | Ramipril | Hypotension | Animal | Monitor |
| Ginger | Clopidogrel | Increased risk of bleeding | Human | Monitor |
| Ginger | Lercanidipine | Hypotension | Theoretical | Monitor |
| Glycyrrhiza glabra | Amlodipine | Hypotension | Human | Not recommended |
| Glycyrrhiza glabra | Simvastatin | Increased drug blood level | Animal | Not recommended |
| Glycyrrhiza uralensis | Bortezomib | Increased drug blood level | Theoretical | Not recommended |
| Grifola | Bisoprolol | Hypotension | Theoretical | Monitor |
| Grifola | Ramipril | Hypotension | Theoretical | Monitor |
| Gymnema | Glyxambi | Hypoglycemia | Theoretical | Monitor |
| Gymnema | Metformin | Hypoglycemia | Theoretical | Monitor |
| Gymnema | Insulin | Hypoglycemia | Theoretical | Monitor |
| Gymnema | Amitriptyline | Impairment of drug effect | Animal | Monitor |
| Gymnema | Losartan | Impairment of drug effect | Animal | Monitor |
| Gymnema | Omeprazole | Impairment of drug effect | Animal | Monitor |
| Gymnema | Oxycodone | Impairment of drug effect | Animal | Monitor |
| Humulus lupulus | Estradiol | Impairment of drug effect | In vitro | Monitor |
| Licorice | Fluticasone | Impairment of drug effect | Animal | Monitor |
| Lycium barbarum | Atorvastatin | Increased drug blood level | Animal | Monitor |
| Lycium barbarum | Amlodipine | Hypotension | Theoretical | Monitor |
| Lycium barbarum | Ramipril | Hypotension | Theoretical | Monitor |
| Matricaria recutita | Duasteride | Increased drug blood level | In vitro | Monitor |
| Matricaria recutita | Esomeprazole | Increased drug blood level | In vitro | Monitor |
| Matricaria recutita | Oxybutynin | Increased drug blood level | In vitro | Monitor |
| Matricaria recutita | Estradiol | Antiestrogen activity | In vitro | Monitor |
| Olive leaf | Bisoprolol | Hypotension | Theoretical | Monitor |
| Olive leaf | Ramipril | Hypotension | Theoretical | Monitor |
| Paeonia lactiflora | Bortezomib | Increased drug blood level | Theoretical | Not recommended |
| Paeonia lactiflora | Aspirin | Increased risk of bleeding | Theoretical | Not recommended |
| Paeonia suffruticosa | Nortitriptylin | Increased drug blood level | In vitro | Monitor |
| Paeonia suffruticosa | Aspirin | Increased risk of bleeding | Theoretical | Monitor |
| Panax ginseng | Bortezomib | Increased drug blood level | Theoretical | Not recommended |
| Panax ginseng | Aspirin | Increased risk of bleeding | Animal | Monitor |
| Polygonum multiflorom | Amlodipine | Increased drug blood level | Theoretical | Monitor |
| Polygonum multiflorom | Venetoclax | Increased drug blood level | Theoretical | Monitor |
| Poria cocus | Insulin | Hypoglycemia | Theoretical | Monitor |
| Rahmanniae glutinosae | Bisoprolol | Hypotension | Animal | Monitor |
| Rahmanniae glutinosae | Ramipril | Hypotension | Animal | Monitor |
| Reishi | Aspirin | Increased risk of bleeding | Theoretical | Monitor |
| Reishi | Bisoprolol | Hypotension | Theoretical | Monitor |
| Schisandra | Tenofovir | Increased drug blood level | Animal | Not recommended |
| Scutelaria baic | Esomeprazole | Increased drug blood level | In vitro | Monitor |
| Scutelaria baic | Nortitriptylin | Increased drug blood level | In vitro | Monitor |
| Scutelaria baic | Apixaban | Increased risk of bleeding | Animal | Monitor |
| Scutelaria baic | Bisoprolol | Hypotension | Animal | Monitor |
| Scutelaria baic | Cadesartan | Hypotension | Animal | Monitor |
| Scutelaria baic | Ramipril | Hypotension | Animal | Monitor |
| Scutelaria baic | Lercanidipine | Hypotension | Animal | Monitor |
| Serenoa serulata | Aspirin | Increased risk of bleeding | Theoretical | Monitor |
| Silybum marianum | Metformin | Hypoglycemia | Theoretical | Monitor |
| Silybum marianum | Venetoclax | Increased drug blood level | Theoretical | Not recommended |
| Tanacetum parthenium | Dexamethasone | Increased drug blood level | Theoretical | Monitor |
| Tanacetum parthenium | Aspirin | Increased risk of bleeding | Theoretical | Not recommended |
| Thyme | Tiotropium bromide | Increased anticholinergic effect | In vitro | Monitor |
| Trigonella | Insulin | Hypoglycemia | Theoretical | Monitor |
| Trigonella | Metformin | Hypoglycemia | Theoretical | Monitor |
| Trigonella | Aspirin | Increased risk of bleeding | Theoretical | Monitor |
| Vitex trifolia | Mirena | Impairment of drug effect | Theoretical | Monitor |
| Withania somnifera | Mirena | Hepatotoxicity | Theoretical | Monitor |
| Ziziphus spinose | Estradiol | Decreased drug blood level | Theoretical | Monitor |
